# Histone deacetylase activity is decreased in peripheral blood monocytes in patients with COPD

**DOI:** 10.1186/1476-9255-9-10

**Published:** 2012-03-23

**Authors:** Yanwei Chen, Ping Huang, Wen Ai, Xiaoli Li, Wei Guo, Jingnong Zhang, Jiong Yang

**Affiliations:** 1 Department of Respiratory Medicine, ZhongNan Hospital of Wuhan University, Wuhan, Peoples Republic of China; 2 Department of Respiratory Medicine, Nanshan Affiliated Hospital of Guangdong Medical College, Guangdong 518052, Peoples Republic of China

**Keywords:** Chronic obstructive pulmonary disease, Histone deacetylase, Cigarette smoke, Inflammation

## Abstract

**Background:**

Histone deacetylase (HDAC) is an enzyme that regulates chromatin structure and inflammatory gene expression. In patients with chronic obstructive pulmonary disease (COPD), while accumulating evidence indicates that the activity of HDAC is decreased in lung tissue alveolar macrophages, HDAC activity in peripheral inflammatory cells has not yet been evaluated in detail.

**Methods:**

HDAC activities in peripheral blood mononuclear cells (PBMC) were investigated in patients with stable COPD (n = 26), non-smoking controls (n = 13), and smoking controls (n = 10), respectively. HDAC activity was measured using an HDAC Activity/Inhibitor Screening Assay Kit. Serum interleukine-8 (CXCL8) levels were determined by ELISA techniques. Lung function test was carried out according to the ATS/ERS guidelines.

**Results:**

Compared with healthy non-smokers, HDAC activity in the PBMCs of COPD patients was decreased by 40% (13.06 ± 5.95 vs. 21.39 ± 4.92 (μM/μg), p < 0.001). In patients with COPD, HDAC activity was negatively correlated to smoke intensity (r = -0.867, p < 0.001). In COPD patients who had smoked for more than 40 pack-years, HDAC activity in PBMC was 40% lower than that in COPD patients who had smoked fewer than 40 pack-years.

Moreover, serum CXCL8 levels in patients with COPD were significantly higher than that in controls and were negatively correlated to HDAC activities.

**Conclusion:**

In patients with COPD, HDAC activity in the PBMCs is lower than that in healthy controls. The reduction of HDAC activity may be associated with smoking exposure through inflammatory pathways.

## Introduction

Chronic obstructive pulmonary disease (COPD) is not only an airway disease but also a systemic disorder that involves systemic inflammatory manifestations [1,2]. In COPD, systemic inflammation is evidenced by increased levels of inflammatory cytokines in circulation even when the condition is stable [3-6]. Inflammatory cells such as macrophages and monocytes have been well known to be involved in pulmonary and systemic inflammation, but their exact role in the pathogenesis of COPD has not yet been clarified [7-10].

Histone acetylase (HAT) and histone deacetylase (HDAC) are families of enzymes that regulate chromatin structure and inflammatory gene expression. Compared to healthy controls, COPD patients showed low HDAC activity in their alveolar macrophages [11,12]. This is believed to be associated with the severity of limitations in air flow. Recently it was found that corticosteroids can synergistically stimulate CXCL8 (a proinflammatory cytokine) production from macrophages [13] and enhances the release of CXCL8 from plasmacytoid dendritic cells [14]. However, no further data has been collected to demonstrate whether peripheral HDAC activity, e.g. that in peripheral blood mononuclear cells (PBMCs), is decreased as well. If HDAC activity is reduced peripherally, then it is interesting to know whether the HDAC activity is related to smoke or even relevant to limitations in air flow.

In this study, we compared HDAC activity in PBMCs from COPD patients and healthy controls. We further explored the associations of HDAC activity with smoking and inflammatory marker.

## Materials and methods

### Subjects

Study subjects were recruited at Renmin Hospital of Wuhan University in Wuhan, China. Criteria for recruiting patients with COPD included the following: 1) Diagnosis of COPD according to GOLD criteria with a ratio of post-bronchodilator forced expiratory volume in 1 second (FEV1)/forced vital capacity (FVC) ≤70% [13]; 2) age over or equal to 40 years; 3) cigarette smoke intake equal to or greater than 10 pack-years; and 4) chest CT scan indicating emphysematous abnormalities, such as barrel chest or central-lobular or pan-lobular emphysema. Exclusion criteria were as follows: 1) The presence of any other structural or functional lung disease, such as a past or current diagnosis of allergic rhinitis or atopy; 2) respiratory tract infection or exacerbation of COPD exacerbation no more than 6 weeks prior to screening; 3) asthma; 4) blood eosinophil count > 600 cell/mm^3^; 5) hospitalization for an acute COPD exacerbation no more than 3 months prior to screening; 6) clinically significant respiratory disease other than COPD; and 7) unstable cardiac condition. Inhaled salbutamol was permitted as needed so long as it was discontinued 24 hours prior to each study visit. Inhaled corticosteroids and oral sustained-release theophyllines were also permitted. Oral or parenteral corticosteroids at maximal doses equivalent to 10 mg/day of prednisone or 20 mg every other day were permitted. Patients who were already on oxygen therapy were included so long as their intake was < 15 hours per day and had been stable for at least 4 weeks prior to screening.

Recruitment criteria for control smokers were as follows: Voluntary cigarette intake of at least 10 pack-years with no history of lung disease or unstable cardiac conditions. The study also included healthy individuals with no history of smoking.

The studies were conducted in accordance with the Declaration of Helsinki, International Conference on Harmonisation Good Clinical Practice Guidelines. The study was approved by the Ethics Committee of the Wuhan University School of Medicine. All participants agreed to participate in this study and provided written informed consent before participating in the study.

### Study design

We performed a prospective study with two visits on separate days. At visit 1, subjects provided written informed consent and were asked about their demographic information, respiratory symptoms by trained research nurse or doctor using standardized respiratory questionnaire. Physical examinations were performed at this time. FEV1 and FEV1 reversibility were tested. Smoking history information was also collected and quantified as pack-years. After visit 1, participants were asked to stop using all drugs and treatment. At visit 2, scheduled at least 2-4 days after visit 1, subjects underwent a spirometry post-bronchodilator test. Peripheral venous blood (20 ml) was extracted from each patient with a heparin syringe. The blood samples were isolated by density centrifugation within 4 hours, then PBMCs were stored at -70°C and serum were stored at -20°C, all samples were prepared less 3 month before analysis.

### Spirometry

Spirometry tests were conducted according to ATS/ERS standardization guidelines for the performance of spirometry and assessment of lung volume using body plethysmography [15]. Typical practice in our laboratory incorporates quality control measures, including daily instrument calibration and review of study quality by a supervising technician and physician prior to interpretation. Flow-volume curves, FVC, relative FVC, FEV1, relative FEV1, and FEV1/FVC were measured using a spirometer (Vmax 229, Sensor-Medics, U.S.) and the best volume of the three manoeuvres was selected for data analysis. Data was expressed as the percentage of predicted normal values. Lung function test was conducted after inhaling 200 μg sabutamol (Glaxo Welcome, Chongqing, China).

### Separation of human PBMCs

Venous blood (20 ml) collected from each subject's ulnar vein was diluted 1:1 with sterile Hank's Balanced Salt Solution. PBMCs were isolated from the blood by density centrifugation as described previously [16]. Diluted venous blood (40 mL) was added on top of 20 ml of LymphoPrep (density, 1.077 g/ml) (Projen Biotechnik, Germany) and centrifuged for 20 minutes at 1,100 g at room temperature. The interface that contained the PBMC was collected and washed twice with phosphate-buffered saline (PBS). Cells were pelleted by centrifugation at 250 g for 8 min and stained with Kimura dye for determination of total cell number. Cell viability, as determined by trypan blue, was uniformly ≥95%.

### Direct extraction of histones from human PBMC

Histones were extracted from PBMC nuclei using HCl and H_2_SO_4 _at 4°C following the method described by Ito [17]. Cells were microcentrifuged for 5 min and the cell pellets were extracted with ice-cold lysis buffer (10 mM Tris·HCl, pH 6.5/50 mM sodium bisulfite, 1% Triton X-100·10 mM MgCl_2_/8.6% sucrose complete protease inhibitor mixture (Roche Molecular Biochemicals) for 20 min at 4°C. The pellet was washed repeatedly in buffer until the supernatant was cleared (centrifuged at 7,500 g × 5 min after each wash). The nuclear pellet was washed in nuclear wash buffer (10mMTris·HCl/13mMEDTA, pH 7.4) and resuspended in 50 μl of 0.2 M HCl and 0.2 M H_2_SO_4_. The supernatant was mixed with 1 ml of ice-cold acetone and left overnight at -20°C. The sample was microcentrifuged for 10 min, washed with acetone, dried, and diluted in distilled water. The protein concentrations of the histone-containing supernatant were determined using a Bradford protein assay kit (Bio-Rad).

### Measurement of HDAC activity in PBMCs

HDAC activity in PBMC nuclear extracts was assessed using an HDAC Activity/Inhibitor Screening Assay Kit (Cayman, U.S.). An acetylated lysine substrate is incubated with samples with HDAC activity. Deacetylation sensitizes the substrate such that treatment with the HDAC developer in the second step releases a fluorescent product. The assay was performed exactly as recommended by the manufacturer. Fluorescence signal was detected at 460 nm using a fluorometric plate reader.

### Analysis of CXCL8 levels in peripheral blood

Serum levels of interleukine-8 (CXCL8) were measured by sandwich ELISA (Jingmei Biotech Co., Ltd., Shenzhen, China) according to the manufacturer's instructions.

### Statistics

Data are expressed as means ± SE (Medians). Analysis of variance was performed with the use of the non-parametric Kruskal-Wallis test. When the result was significant, the Mann-Whitney U test was performed for comparisons between groups (SPSS software version 15.0). Correlation coefficients were calculated with the use of Spearman's rank method. *P *< 0.05 was considered to indicate statistical significance. All reported P values are two-sided.

## Results

### Patient characterization

Demographic data from all participants are presented in Table 1. There were 23 healthy volunteers, among them 10 were habitual cigarette smokers and 13 were never smokers. All 26 patients with COPD were habitual cigarette smokers, of whom 7 were in GOLD stage 1, 8 in GOLD stage 2, 8 in stage 3, and 3 in stage 4 (Table 1,2). No significant differences were observed between COPD cases and controls in terms of age, height, and body weight.

**Table 1:** Baseline characteristics of the Study Subjects *

	COPD group (n = 26)	Healthy smoker group (n = 10)	Healthy non-smoker group (n = 13)
Height (cm)	164.54 ± 6.08 (165)	167.56 ± 6.60 (170)	169.54 ± 5.94 (169)
Age (year)	63.50 ± 19.75 (71)	52.70 ± 12.33 (51)	54.85 ± 11.42 (54)
Weight (kg)	63.31 ± 11.61 (61)	68.50 ± 14.49 (74)	68.00 ± 7.92 (70)
FVC_volume (L)	2.68 ± 0.92 (2.36)	4.09 ± 0.68 (4.01)	3.92 ± 0.72 (3.82)
FVC%	86 ± 20 (89)	121 ± 21 (116)	113 ± 21 (114)
FEV1_volum (L)	1.35 ± 0.63 (1.20)	3.34 ± 0.55 (3.38)	3.00 ± 0.77 (2.90)
FEV1%	55 ± 19 (50)	124 ± 21 (115)	109 ± 27 (112)
FEV1/FVC%	50 ± 12 (50)	82 ± 5 (80)	76 ± 6 (79)
Smoking(pack-years)	33.46 ± 17.19(30)	36.5 ± 18.11(35)	0
Sex (M/F)	21/5	8/2	9/4
White blood cell counts in peripheral blood(10^9^)	7.12 ± 2.56(6.95)	7.06 ± 2.44(6.90)	6.88 ± 2.66(6.73)
Medication (No)			
Oral corticosteroid	0	0	0
Inhaled corticosteroid	3	0	**0**
Short-acting beta-adrenergic-receptor agonist	4	0	**0**
Theophyline	0	0	**0**
Oxygen	1	0	**0**

*COPD denotes chronic obstructive pulmonary disease, FEV 1 forced expiratory volume in one second, FVC forced vital capacity, pack-years the number of cigarettes smoked per day, or 20 cigarettes, multiplied by the number of years of smoking. Plus-minus values are means ± SE(Mediam)

**Table 2:** Baseline characteristics of the COPD subgroup Subjects *

	Smoke ≧ 40 pack-year(n = 13)	Smoke < 40 pack-year(n = 23)
Height (cm)	163.00 ± 6.02(163.00)	166.52 ± 6.17(167.00)
Age (year)	68.31 ± 11.11(74.00)	56.09 ± 20.50(61.00)
Weight (kg)	66.08 ± 12.70(60.00)	64.00 ± 12.58(65.00)
FVC_volume (L)	2.73 ± 0.96(2.36)	3.26 ± 1.10(3.38)
FVC%	92 ± 22(95)	98 ± 27(100)
FEV1_volum (L)	1.62 ± 0.84(1.32)	2.06 ± 1.20(1.43)
FEV1%	70 ± 31(63)	76 ± 41(59)
FEV1/FVC%	45.80 ± 30.02(30.00)	48.21 ± 24.77(32.40)
Smoking(pack-years)	53.85 ± 7.68(60.00)	23.26 ± 9.37(30.00)
Sex (M/F)	11/2	20/3
Medication (No)		
Oral corticosteroid	0	0
Inhaled corticosteroid	2	1
Short-acting beta-adrenergic-receptor agonist	2	2
Theophylline	0	0
Oxygen	1	0

*COPD denotes chronic obstructive pulmonary disease, FEV 1 forced expiratory volume in one second, FVC forced vital capacity, pack-years the number of cigarettes smoked per day, or 20 cigarettes, multiplied by the number of years of smoking. Plus-minus values are means ± SE(Mediam)

### HDAC activity in PBMCs of COPD patients

**Figure 1:**
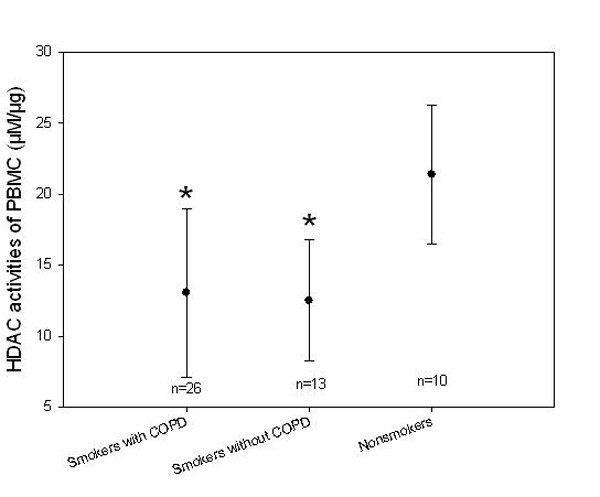
**HDAC activities of PBMC (μM/μg) between smokers and non-smokers**. HDAC activities of PBMC in both smokers with COPD and smokers without COPD were significantly lower than that in non-smokers. (* p < 0.01 compared to Non-smokers).

HDAC activity in the PBMCs of COPD patients was significantly decreased by 40% as compared to that in healthy non-smokers (13.06 ± 5.95 vs. 21.39 ± 4.92 μM/μg, *p *< 0.001). HDAC activity in PBMCs of healthy smokers was also 40% lower than that observed in healthy non-smokers (12.50 ± 4.27 vs. 21.39 ± 4.92 μM/μg, *p *< 0.001). (Figure 1)

### Relationship between HDAC activity in PBMCs and cigarettes smoking levels

**Figure 2:**
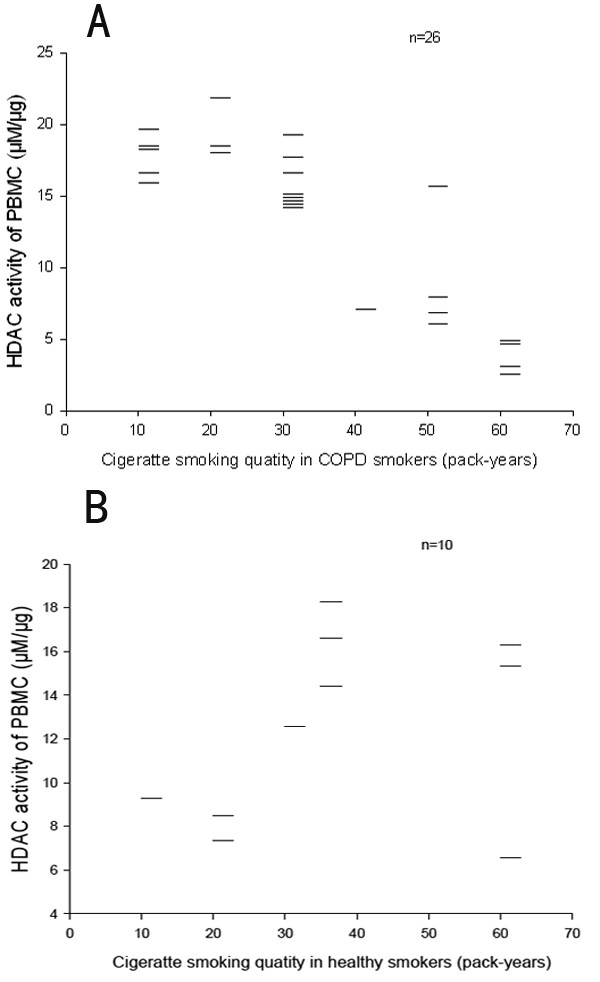
**Relationship between HDAC activity and smoking exposure levels**. In heavy-smoking COPD patients (≥ 40 pack-years), HDAC activity in the PBMCs was lower than that in patients smoked fewer than 40 pack-years.

**Figure 3:**
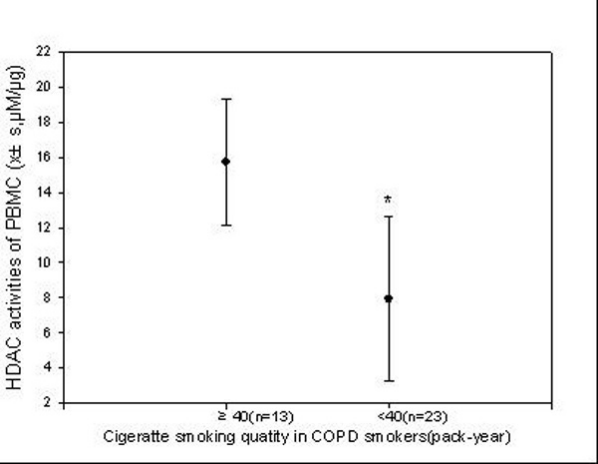
**Relationship between smoking levels and serum CXCL8 concentrations in COPD smokers**. At 40 pack-years, increases in blood CXCL8 levels became statistically significant.

HDAC activity in PBMCs was decreased as the smoking levels (pack-years) increased, (Figure 2). In COPD patients who were heavy smokers (≥ 40 pack-years), HDAC activity in the PBMCs was 40% lower than that in COPD patients who smoked fewer than 40 pack-years(Figure 3).

### CXCL8 levels in peripheral blood

**Figure 4:**
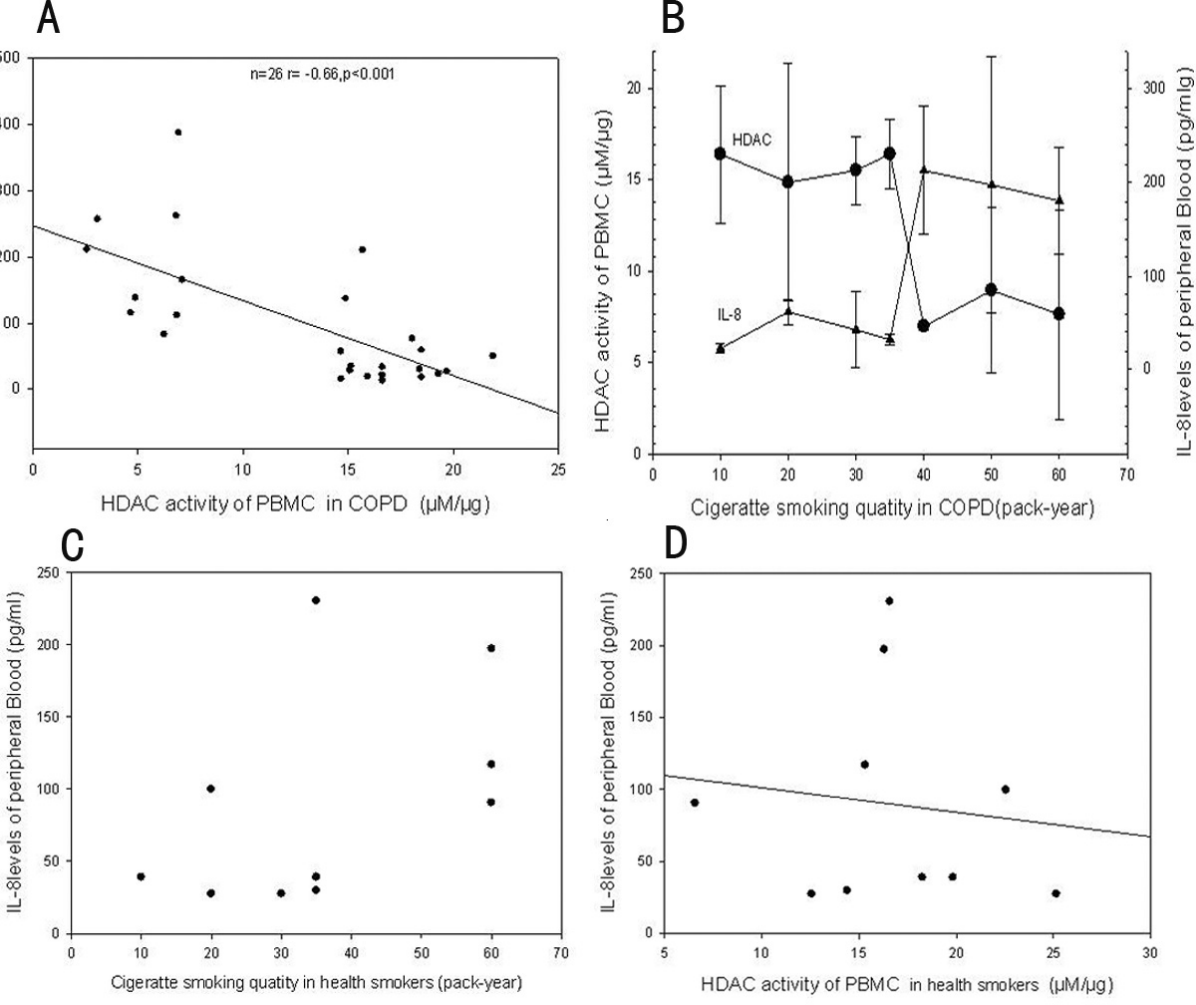
**Correlation of smoking quantity with HDAC activity in COPD patients**. When smoking quantity reaches 40 pack-years, HDAC activity decreased significantly (* p < 0.01 compared to Non-smokers).

Higher serum CXCL8 levels were found in stable COPD patients than in non-smoker controls. In addition, increased serum CXCL8 levels were negatively correlated to HDAC activity in PBMCs (r = -0.468, p < 0.01) (Figure 4). Patients who smoked more than 40 pack-years had higher serum CXCL8 levels than those who had smoke less than 40 pack-years.

### Relationship between HDAC and airflow limitation

No correlation was found between the severity of airway obstruction and HDAC activity or serum CXCL8 level (all p > 0.05).(Table 3).

**Table 3:** Correlation of HDAC activity in PBMC with Lung Function (n = 26)

		HDAC	FVC (L)	FVC (%)	FEV1 (L)	FEV1 (L)	FEV1/FVC (%)
HDAC	r		0.164	0.234	0.249	0.294	0.057
	p	.	0.423	0.249	0.221	0.144	0.783

## Discussion

In the present study, we show that HDAC activity in PBMCs was lower in stable COPD patients than that in healthy non-smoker controls. The decrease of HDAC activity was significantly associated with smoking exposure levels and serum CXCL8 concentrations, suggesting that HDAC activity in PBMC may be modified by systemic inflammation and smoking quantities.

Our findings are consistent with previous observations that systemic inflammation is implicated in the development of COPD [2].The origins of the systemic inflammation in patients with COPD are not entirely clear. It is likely that the systemic inflammation is a consequence of spillover of inflammatory mediators from the lungs to the systemic compartment [18-20]. Decreased HDAC activity was observed in bronchial biopsies and alveolar macrophage from subjects with COPD [12]. Together with our observation, it is clear that HDAC activity was decreased not only in the lung tissue and alveolar cells but also in the PBMCs. Interestingly, in heavy-smoking COPD patients (≥ 40 pack-years), HDAC activity in the PBMCs was 40% lower than that in patients smoked fewer than 40 pack-years.

It has been widely accepted that smoking-induced inflammation plays a critical role in the pathogenesis of COPD [21]. For instance, sustained inflammation exists in ex-smokers as well as in current smokers in COPD [22-24]. Our results suggest that the number of cigarettes smoked over time may be related to serum levels of CXCL8, a marker of systemic inflammation. Our results also show that HDAC activity in PBMCs is decreased as the cigarette smoking levels (pack-years) over 40, suggesting heavy smokers seem to have a far more significant reduction of HDAC activities and increased CXCL8 than light smokers. This observation may partially explain why sustained inflammation exists in ex-smokers as well as in current smokers in COPD

Our results are also in agreement with prior reports that serum CXCL8 levels are increased in stable COPD patients [3,25], supporting previous findings that COPD is a systemic inflammatory disease. Furthermore, we found that increased serum CXCL8 levels were negatively correlated with HDAC activities in PBMCs, suggesting that HDAC has a role in systemic inflammation of stable COPD patients.

In this study, no correlation was found between the severity of airway obstruction and HDAC activity or serum CXCL8 level. This suggests that systemic inflammation may not directly contribute to COPD development but by cooperation with other biological pathways.

There are a few limitations in this study. Firstly, the sample size was relatively small. Thus we were not able to conduct stratified analysis. Second, HAT activity was not analyzed to determine whether the decreased HDAC activity was related to HAT activity. Third, we did not analyze local lung inflammation simultaneously. Thus, the relationship between systemic inflammation and lung local inflammation cannot be defined. Finally, because all the COPD patients in our district are almost smokers, we couldn't enroll COPD patients due to occupational exposures, including organic and inorganic dusts and chemical agents and fumes, which will give more information about the HDAC activity of PBMC in non-smoking COPD patients.

## Conclusion

In conclusion, our findings suggest that smoking is associated with decreased HDAC activity and high levels of CXCL8 in patients with COPD. Further studies including more individuals are required to confirm our results and to explore detailed mechanisms underlying the associations between HDAC activities and smoking levels in COPD.

## Abbreviations

COPD: Chronic obstructive pulmonary disease; HDAC: Histone deacetylase; PBMC: Peripheral blood mononuclear cells; HAT: Histone acetylase; CXCL8: Interleukin-8; GOLD: Global initial of obstructive pulmonary disease; PBS: Phosphate-buffered saline; ATS: American Thoracic Society; ERS: European Respiratory Society; FEV 1: Forced expiratory volume in one second; FVC: Forced vital capacity; ELISA: Enzyme linked immunosorbent assay; SPSS: Statistical Product and Service Solutions.

## Competing interests

The authors declare that they have no competing interests.

## Authors' contributions

YC and PH participated in the experimental work and drafting the manuscript. WA, XL and WG participated in the experimental work. JY and YC participated in the study design and drafting of the manuscript. JZ conceived the study, participated in its design and coordination and drafting of the manuscript. All authors read and approved the final manuscript.
